# The influence of type of consultation and patient characteristics on non-attendance at adult ADHD consultation

**DOI:** 10.1192/bjb.2025.35

**Published:** 2026-04

**Authors:** Paul Stephenson, Marianne Durand, Matthew Humphreys, Alex Stewart

**Affiliations:** 1 Consultant Psychiatrist, Adult Autism Service and Adult ADHD Service, Cheshire and Wirral Partnership NHS Foundation Trust, Chester, UK; 2 Consultant Clinical Psychologist, Community Learning Disabilities Team, Cheshire and Wirral Partnership NHS Foundation Trust, Chester, UK; 3 Business and Project Manager/Research Grant Lead, Research, Effectiveness, Academic, Learning (REAL), Cheshire and Wirral Partnership NHS Foundation Trust, Chester, UK; 4 Honorary Senior Research Fellow, College of Life & Environmental Science, University of Exeter, UK

**Keywords:** DNA, virtual, attendance, patient factors, service factors

## Abstract

**Aims and method:**

Non-attendance at out-patient appointments of adult attention-deficit hyperactivity disorder (ADHD) services incurs significant costs and contributes to lost service provision and unmet clinical needs. This cross-sectional study of clinical contacts, between 1 July 2022 and 30 June 2023 in a specialist adult ADHD service, aimed to identify factors, including type of consultation, associated with non-attendance.

**Results:**

Of 3673 organised clinic appointments, 2815 (77%) were attended: 2314 (82%) by telephone and 501 (18%) as in-person appointments; non-attendance rates were 17 and 42%, respectively. Patient characteristics associated with improved attendance included: female gender, age >30 years and presence of other psychiatric diagnoses.

**Clinical implications:**

This study will assist adult ADHD service providers to maximise patient attendance. The role of telephone (or virtual) clinics must be considered. Enhanced appointment reminders and improving access to services targeting at-risk groups could also improve attendance rates.

Attention-deficit hyperactivity disorder (ADHD) is a neurodevelopmental disorder characterised by persistent and impairing inattention and/or hyperactivity/impulsivity.^1^ ADHD has a worldwide prevalence of 2–4% in adults;^2,3^ prevalence in high- and upper-middle-income countries is 3.6%.^2^ ADHD usually presents in childhood and persists into adulthood in approximately 65% of cases.^4,5^

Patients with ADHD have been found to be more likely to miss out-patient appointments compared with those with other psychiatric disorders.^6^ This may be related to the core symptoms of ADHD, which include disorganisation and forgetfulness. National Health Service (NHS) England reported an overall non-attendance rate of 7.6% in out-patient clinics during 2021/22; the importance of increased out-patient attendance across all healthcare specialties towards improving patient experience, freeing up capacity and treating long-waiting patients has been emphasised.^7^ This is a fundamental issue for adult ADHD services across the UK, given the pressure on services and long waiting lists for diagnosis and treatment.^8^

The factors associated with patient attendance have been explored across multiple healthcare settings.^9^ Sociodemographic factors have been found to be associated with patient attendance rate. A consistent pattern is reported of higher levels of attendance among older patients^10,11^ and females.^12,13^ There is evidence to suggest that social deprivation is associated with lower rates of clinic attendance; however, the association between ethnicity and attendance rate is inconsistent.^9^ The time of year of appointment may be associated with patient attendance rate: more missed appointments have been reported during winter months.^14,15^

More recently, the social restrictions imposed during the COVID-19 pandemic necessitated a novel approach to out-patient care across all areas of healthcare; many services opted to introduce virtual clinics, which involved telephone and/or video assessments with variable clinical results and patient satisfaction.^16,17^ It has been suggested that ADHD is a disorder that is particularly suited to virtual clinics.^18^ A recent systematic review investigating the effectiveness of virtual clinics in the treatment of ADHD suggests that it may be a viable method to provide assessment and evidence-based pharmacologic treatment.^19,20^

The Cheshire and Wirral Partnership NHS Foundation Trust (CWP) adult ADHD service has longstanding difficulties with non-attendance of patients invited to out-patient clinics. The service operated primarily using telephone clinics during the COVID-19 pandemic. It was noted anecdotally that patient attendance may have improved when using telephone appointments during this time. The service has more recently facilitated a gradual return to in-person clinics following the easing of social restrictions associated with the COVID-19 pandemic; the adult ADHD service in this study is currently delivered through a hybrid of in-person and telephone appointments. However, the issue of non-attendance at appointments remains a concern for the CWP adult ADHD service; this phenomenon continues to contribute to lost service provision and unmet clinical needs in the patient group. Non-attendance at out-patient appointments incurs significant costs.^7^

This study aimed to improve understanding of patients’ sociodemographic and clinical factors, as well as service delivery factors, to determine which of these impact patient attendance.

## Method

### Study setting

The CWP adult ADHD service is a specialist service that provides assessment, diagnosis and treatment of ADHD. The service is available to adults registered with a general practitioner in the boroughs of Wirral, Cheshire West and Chester, Cheshire East and the City of Liverpool. Patients are reviewed in the clinic by either medical (psychiatrists) or non-medical clinicians (nurse prescribers). Patients are prioritised for assessment based on the length of time spent on the service’s waiting list; occasionally a patient can be prioritised based on clinical need under guidance from the clinical team. Patients are mostly seen on a ‘first come, first served’ basis, with allocation to the next available clinician, whether that is an in-person or telephone consultation. The service’s dedicated administration team invite patients to attend clinic by mail at least 7 days before the date of appointment; patients are advised to contact the service to rearrange the appointment if unable to attend. A reminder text message is sent to the patient one working day before the date of appointment. Previous research has shown that text reminders can increase clinic attendance rate by up to 50%, compared with no appointment reminder.^21,22^

### Data collection

This cross-sectional study design, using secondary data, analysed attendance details of patients invited for consultation under the CWP adult ADHD service between 1 July 2022 and 30 June 2023. This period represents the time during which the service reliably delivered a hybrid service, with both telephone and in-person appointments offered. A continuous 12-month period was considered suitable because this accounted for possible seasonal effects influencing patient attendance at the clinic.

Anonymised data on the patients’ electronic health records were extracted for each scheduled consultation:

- demographic variables: age at date of appointment, gender, ethnicity, postcode (to derive Index of Multiple Deprivation (IMD) score)
- clinical variables: other psychiatric diagnoses as recorded by ICD-10 coding^23^
- service variables: contact method (telephone or in-person), appointment type (initial or review), date of appointment, treating clinician (medical or non-medical practitioner).

Data were analysed using the Statistical Package for the Social Sciences (SPSS v.28). Categorical variables in respect of patients who did and did not attend appointments were compared using unadjusted odds ratios; variables with *P* < 0.1 were further analysed by multivariate modelling (logistic regression).

### Ethical statement

This project was approved by the Clinical Directors and Research and Development team at CWP. The study analysed retrospective data, which were collected routinely as part of standard clinical care. Staff had access to these data as part of their clinical duties. All data were anonymised and were not used for any purpose other than that for which they were collected; there were therefore no ethical or confidentiality issues requiring NHS research approvals. This study was not defined as research, according to the Health Research Authority algorithm (https://www.hra-decisiontools.org.uk/research/), and was reviewed and approved in the same way as evaluations.

## Results

There were 3673 planned appointments with the CWP adult ADHD service between 1 July 2022 and 30 June 2023; patients did not attend appointments in 858 (23.4%) of cases.

Demographic variables were associated with appointment attendance rate (Table 1); specifically, patients aged <30 years had a non-attendance rate higher than those aged ≥30 years, and males had a higher non-attendance rate than females. Deprivation index had no significant association with attendance rate. A third of ethnicity data were incomplete, and therefore ethnicity was not included in the analysis.

**Table 1:** Characteristics of patients who did and did not attend appointments with the adult ADHD service, and selected characteristics of their appointments

Variable	Attended	Did not attend	Non-attendance rate (%)	P*
Number	2815	858	23.4	
Demographic variables				
Age (years)				
Mean (s.d.)	30.80 (11.26)	28.59 (10.83)		**<0.001
<20	481	193	28.6	0.022
20–29	184	81	30.6	
30–39	1020	318	23.8	
40–49	574	133	18.8	
>50	556	133	19.3	
Gender				
Female	1034	259	20.0	<0.001
Male	1781	599	25.2	
IMD^a^				
Quintile 1	1651	527	24.2	0.080
Quintile 2	416	125	23.1	
Quintile 3	277	71	20.4	
Quintile 4	262	80	23.4	
Quintile 5	183	50	21.5	
Ethnicity				
White	1855	543	29.3	
Black and minority ethnic	143	30	21.0	
Unknown	866	285	32.9	
Clinical variables				
Previous psychiatric diagnosis^b^				
Number of diagnoses				
0	760	309	28.9	<0.001
1	1016	263	20.6	
2	708	190	21.2	
>2	331	96	22.5	
Service characteristics				
Contact method				
Face-to-face	501	369	42.4	<0.001
Telephone	2314	489	17.4	
Consultation type				
Follow-up	1860	514	21.7	0.034
Initial	873	334	27.7	
Unknown	82	10	10.9	
Date of appointment				
1 January to 31 March	601	243	28.8	<0.001
1 April to 30 June	847	205	19.5	
1 July to 30 September	673	198	22.7	
1 October to 31 December	694	212	23.4	
Clinician type				
Doctor	729	215	22.8	0.620
Non-medical prescriber	2086	643	23.6	

ADHD, attention-deficit hyperactivity disorder.

a. Index of multiple deprivation (IMD) is a measure of relative deprivation for small, fixed geographic areas of the UK. IMD classifies these areas into five quintiles based on relative disadvantage, with quintile 1 being the most deprived and quintile 5 the least.

b. ICD classification of psychiatric diagnoses is shown in Table 2.

*Statistical significance calculated using chi-squared test unless otherwise specified.

**Statistical significance calculated using one-way analysis of variance.

Patients with no recorded psychiatric diagnosis were significantly associated with reduced attendance rate (Table 1). Patients with an existing F90–F98 psychiatric diagnosis (behavioural and emotional disorders with onset usually occurring in childhood and adolescence) had a higher attendance rate than those without such a confirmed diagnosis (F90–F98 includes the diagnosis of ADHD). Other specific psychiatric diagnoses were not significantly associated with attendance rate (Table 2).

**Table 2:** Previous psychiatric diagnoses of patients who did and did not attend appointments with the adult ADHD service

Psychiatric comorbidity	Attended	Did not attend	Non-attendance rate (%)	P*
F00–F09: organic, including symptomatic, mental disorders				
No	2812	857	23.4	0.940
Yes	3	1	25.0	
F10–F19: mental and behavioural disorders due to psychoactive substance use				
No	2636	792	23.1	0.170
Yes	179	66	26.9	
F20–F29: schizophrenia, schizotypal and delusional disorders				
No	2783	846	23.3	0.540
Yes	32	12	27.3	
F30–F39: mood (affective) disorders				
No	2537	792	25.2	0.055
Yes	228	66	22.4	
F40–F48: neurotic, stress-related and somatoform disorders				
No	2952	769	20.7	0.049
Yes	363	89	19.7	
F50–F59: behavioural syndromes associated with physiological disturbances and physical factors				
No	2781	849	23.4	0.710
Yes	34	9	20.9	
F60–F69: disorders of adult personality and behaviour				
No	2648	810	23.4	0.710
Yes	167	48	22.3	
F80–F89: disorders of psychological development				
No	2281	714	23.8	0.150
Yes	534	144	21.2	
F90–F98: behavioural and emotional disorders with onset usually occurring in childhood or adolescence				
No	888	335	27.4	<0.001
Yes	1927	523	21.3	

ADHD, attention-deficit hyperactivity disorder.

*Statistical significance calculated using chi-squared test.

Service delivery variables seen from the perspective of the patient were found to influence attendance rate (Table 1). The rate of non-attendance was 2.5 times higher for in-person appointments than for telephone appointments on univariate analysis. The time of year of the appointment influenced attendance rate: patients were less likely to attend appointments between 1 January and 31 March compared with other 3-month periods. The type of clinician offering the appointment (medical or non-medical practitioner) had no significant impact on attendance rate (Table 1).

Multivariate analysis confirmed that indviduls are more likely to attend telephone appointments compared with in-person appointments (Table 3), and were over three times more likely to miss in-person appointments. Females were more likely to attend appointments. The effects of previous psychiatric diagnosis and time of year also remained significant in the multivariate analysis; this analysis indicated specific clinical scenarios with impact on attendance rate, e.g. a female patient, with two previous psychiatric diagnoses and having telephone contact between April and June had the lowest risk of non-attendance (11%).

**Table 3:** Multivariate model analysis demonstrating odds of attendance at appointment; only significant predictors of attendance are included in the model

Variable	Regression coefficient	Standard error	Odds ratio	95% confidence interval	*P*
Gender					
Female			1.00		
Male	0.32	0.06	1.38	1.16, 1.64	<0.001
Number of psychiatric diagnoses					
0			1.00		
1	−0.38	0.10	0.68	0.56, 0.83	<0.001
2	−0.41	0.11	0.65	0.53, 0.82	<0.001
3 or more	−0.30	0.14	0.74	0.57, 0.98	0.035
Contact method					
Face-to-face			1.00		
Telephone	−1.26	0.09	0.29	0.24, 0.34	<0.001
Date of appointment					
1 January to 31 March			1.00		
1 April to 30 June	−0.34	0.11	0.71	0.57, 0.89	0.003
1 July to 30 September	−0.23	0.12	0.79	0.63, 0.99	0.046
1 October to 31 December	−0.01	0.11	0.74	0.59, 0.92	0.007
Constant	−0.04				0.769

## Discussion

The non-attendance rate at CWP adult ADHD out-patient clinics was three times greater for people invited to in-person clinics compared with those offered telephone consultations. Other important sociodemographic and clinical characteristics increasing non-attendance include male gender, age <30 years and absence of other psychiatric diagnosis.

Non-attendance rates were 17% for telephone and 42% for in-person appointments (multivariate odds ratio 0.29; Table 3). Individuals with ADHD may struggle with the planning and organisation required to attend in-person appointments, due to inattention and distractibility.^6^ Lower attendance rate is known to be related to travelling time and associated costs, and transport links may influence non-attendance; however, our study lacked available data to analyse this possibility. In addition, the clinic environment can be overwhelming for those with neurodevelopmental disorders.

Non-attendance rates in men and women were 25 and 20%, respectively. Males are known to be less likely to attend planned appointments,^4,25^ which may be attributed to their greater self-reliance and a reluctance to seek medical help.^26^ However, previous studies did not fully explore the reasons behind observed gender differences.^9^

Clinic attendance for persons aged <30 years was statistically less likely than for those aged ≥30 years. Previous work has consistently found that younger individuals are more likely to miss appointments.^24,27^ This may be explained by childcare responsibilities, work commitments and better health.^25^ ADHD persists into adulthood in approximately 65% of cases;^4,5^ it is possible that young adults with ADHD are more likely to experience improved symptoms while those aged ≥30 years and with more pervasive symptoms continue to demonstrate more significant symptoms. The referred patient may no longer perceive they need help by the time an appointment is offered, given the long waiting times for assessment.

The clinic attendance rate was lower for patients with no existing psychiatric diagnosis. This may be explained by previous research which has shown that adults with ADHD are often misdiagnosed.^28^ Alternatively, people without additional disease burden may be better able to contain ADHD symptoms; they may place less value on input from a specialist ADHD service. Specific psychiatric conditions were not found to be significantly associated with attendance rate other than behavioural and emotional disorders with onset usually occurring in childhood and adolescence (F90–F98). These (which include ADHD) were associated with a higher attendance. This may be a confounder because it is to be expected that patients who have previously attended a diagnostic assessment with a specialist ADHD service are more likely to attend subsequent reviews.

Most individuals accessing the adult ADHD clinic lived in areas with high levels of deprivation; nevertheless, there was no significant association identified between non-attendance and social deprivation. This is an unexpected finding; previous work suggests that people with high social deprivation are more likely to miss appointments.^24^ However, this association is weak;^9^ it is possible that the size of our study is insufficient to confirm the expected association.

The time of year of the appointment was found to have a statistically significant association with non-attendance rate; people were most likely to miss appointments between January and March. This may be caused by barriers related to winter weather; individuals are more likely to be unwell with respiratory illness during the winter months.^29^ In addition, this study focused on a time period that closely followed the COVID-19 pandemic; some people might have chosen to miss clinic appointments to minimise COVID-19 infection risk, so this may be less of a winter phenomenon and more of a pandemic-related response. Non-attendance rates were broadly constant for the rest of the year.

The consultation type (initial or review) and clinician type (medical or non-medical) had no significant impact on attendance rate. This is not surprising, given that there has been no consistent pattern in previous research. Some studies found attendance rates lower in follow-up appointments, possibly because initial appointments are more likely to be prompted by the patient.^9,30^ However, other studies found attendance rates to be higher for review appointments, possibly related to positive patient–clinician relationships.^31,32^ Previous studies found no consistent association between clinician type and patient attendance.^9^

To our knowledge, this is the first study to explore the characteristics affecting non-attendance of patients at an adult ADHD clinic. Our overall non-attendance rate of 23.4% is consistent with that reported for UK general psychiatry clinics, of between 15 and 22%.^24,33^ There are no previous studies describing attendance at adult ADHD clinics. Our multivariate analysis indicated that the lowest non-attendance rate, based on the studied characteristics, was 11% for a female patient, with two previous psychiatric diagnoses and having telephone contact between April and June; this is higher than the 7.6% average reported by NHS England during 2021/22.^7^

The following limitations of this study should be noted. The quality of the data could have been affected by variation in clinical interpretation and data recording. The focus was on patient consultations, and it is likely that some individuals have been represented on multiple occasions in the analysis; we are unable to quantify this. This study does not identify the reasons for non-attendance at a clinic; the difference in clinical outcome or patient satisfaction related to contact method is not explored. Further qualitative research may improve understanding of patient motivation for attendance or non-attendance to better inform service delivery. The generalisability of our findings may have been affected by the geography of the studied population; there may be significant differences between inner-city urban areas with good transport links in comparison with sparsely populated rural areas with poorer transport links. In-person attendance at ADHD clinics may be higher in densely populated inner-city areas, although this needs further clarification; this study lacked available data to investigate this possibility. The large electronic data-set was a major strength of this study, allowing meaningful multifactorial statistical analysis. The key strength of this study is identification of variables that may influence attendance rate in a clinical specialty where demand for services far outweighs supply. ADHD is common in UK general psychiatric out-patient clinics, being present in approximately 20% of patients;^34^ our findings may be generalisable beyond specialist adult ADHD clinics.

This study may influence the future design of adult ADHD services, to improve patient attendance and outcomes. The resources allocated to adult ADHD services have failed to keep pace with the increasing demand, resulting in lengthy waiting lists for specialist services; waiting times for assessment have been reported at up to 4 years.^8^ There has been an organisational return to in-person clinics following the easing of social restrictions associated with the COVID-19 pandemic. However, this may hinder services aiming to reduce waiting lists and provide treatment for those diagnosed with ADHD. When designing services to ensure optimal use of clinic time, service providers should consider the striking threefold lower non-attendance rates for telephone consultations found in this study. Improved attendance rates may be facilitated by focusing on the demographic subsets most likely to miss appointments (male gender, age <30 years, absence of previous psychiatric diagnosis). Those at highest risk of non-attendance may benefit by having an enhanced reminder service. Multiple notifications are more effective at improving attendance than single notifications, and voice reminders are more effective than text reminders.^21^ Attendance may be improved by allowing individuals to reply to reminder notifications to confirm or cancel the appointment.^7^

Lost clinician time and unused clinical resources due to clinic non-attendance come at a cost.^35^ ADHD services are faced with a demand–capacity mismatch; there is a need to reflect on how best services are configured, and such review and reflection should include consideration of mode of contact, e.g. virtual versus in-person assessments. However, we urge caution when considering the potential benefits of virtual clinics. ADHD services must be designed while taking into account local data and the personal needs of patients for both assessment and monitoring. While satisfaction with virtual out-patient consultation is largely positive,^36,37^ some may struggle with lack of non-verbal cues and have difficulty building therapeutic alliance with their clinician during virtual consultation; carers may feel excluded from virtual consultation.^38^ In-person consultation may be needed to complete physical health monitoring. Follow-up reviews often involve medication titration, which should include physical health monitoring; virtual review assessments are therefore likely to introduce an unintended burden of additional checks in other areas of community care, e.g. general practice, pharmacies.

In conclusion, this study identified that the non-attendance rate at adult ADHD outpatient clinics depends on multiple factors. Most significantly, the contact method had a significant association with non-attendance rate; people were three times more likely not to attend in-person appointments than telephone appointments. Patient characteristics were also associated with reduced patient attendance, including male gender, age <30 years, absence of previous psychiatric diagnosis and time of year (January to March). These findings may help service providers improve patient attendance by considering the most appropriate adult ADHD service design based on identified risk factors for non-attendance; this may have implications for non-attendance in services beyond specialist adult ADHD clinics.

## Data Availability

The data that support the findings of this study are available from the corresponding author, P.S., on reasonable request.
