# The influence of type of consultation and patient characteristics on non-attendance at adult ADHD consultation – ERRATUM

**DOI:** 10.1192/bjb.2025.10127

**Published:** 2026-04

**Authors:** Paul Stephenson, Marianne Durand, Matthew Humphreys, Alex Stewart

In the original publication of this article, there was a typographical error in the title as follows:

‘The influence of type of consultation and patient characteristics on non-attendence at adult ADHD consultation’

This should have been:

‘The influence of type of consultation and patient characteristics on non-attendance at adult ADHD consultation’

The article has been updated to reflect this change, and this correction notice published. The Publisher apologises for this error.
